# Sensitive ADAR editing reporter in cancer cells enables high-throughput screening of small molecule libraries

**DOI:** 10.1093/nar/gky1228

**Published:** 2018-12-22

**Authors:** Kajsa Fritzell, Li-Di Xu, Magdalena Otrocka, Claes Andréasson, Marie Öhman

**Affiliations:** 1 Department of Molecular Biosciences, The Wenner-Gren Institute, Stockholm University, Svante Arrhenius väg 20C, 106 91 Stockholm, Sweden; 2 Chemical Biology Consortium Sweden, Science for Life Laboratory, Department of Medical Biochemistry and Biophysics, Karolinska Institutet, 171 77, Stockholm, Sweden

## Abstract

Adenosine to inosine editing is common in the human transcriptome and changes of this essential activity is associated with disease. Children with *ADAR1* mutations develop fatal Aicardi-Goutières syndrome characterized by aberrant interferon expression. In contrast, ADAR1 overexpression is associated with increased malignancy of breast, lung and liver cancer. ADAR1 silencing in breast cancer cells leads to increased apoptosis, suggesting an anti-apoptotic function that promotes cancer progression. Yet, suitable high-throughput editing assays are needed to efficiently screen chemical libraries for modifiers of ADAR1 activity. We describe the development of a bioluminescent reporter system that facilitates rapid and accurate determination of endogenous editing activity. The system is based on the highly sensitive and quantitative Nanoluciferase that is conditionally expressed upon reporter-transcript editing. Stably introduced into cancer cell lines, the system reports on elevated endogenous ADAR1 editing activity induced by interferon as well as knockdown of ADAR1 and ADAR2. In a single-well setup we used the reporter in HeLa cells to screen a small molecule library of 33 000 compounds. This yielded a primary hit rate of 0.9% at 70% inhibition of editing. Thus, we provide a key tool for high-throughput identification of modifiers of A-to-I editing activity in cancer cells.

## INTRODUCTION

Editing of RNA by deamination of adenosines to inosines (A-to-I) is an essential process in mammals (1–3) that can be catalyzed by two enzymes, ADAR1 and ADAR2. ADAR2 has been shown to be necessary for a functional brain, mainly by editing the A2 subunit (GluA2) of the AMPA glutamate receptor transcript (2). Editing of the Q/R site in the GluA2 mRNA modifies a glutamine codon with the consequence that arginine is incorporated since inosine is read as guanosine by the translational machinery. Nearly all of the GluA2 transcripts are edited at the Q/R site in exon 11 in the healthy human brain, as well as in all other mammalian brains analyzed. In addition, the GluA2 transcript is highly edited at another site in exon 13. This editing event also leads to changed translation form arginine to glycine (R/G). Moreover, several other genes involved in neurotransmission have been shown to utilize A-to-I editing to express alternative protein isoforms with functional consequences for receptor topology and assembly (reviewed in (4)). These transcripts are frequently edited by both ADAR1 and ADAR2, but some sites are enzyme specific (2,5,6). ADAR1 deficient mice die as embryos from hematopoietic defects and liver failure while ADAR2 knockouts exhibit less severe phenotypes, being seizure prone and die a few weeks after birth (1–3,7). The severe phenotype of ADAR1 knockout mice implicates this enzyme in important functions in tissues other than brain. ADAR1 is expressed as two isoforms; a ubiquitously expressed short form (ADAR1^p110^) and an interferon-inducible long form (ADAR1^p150^) (8). Loss of ADAR1 in hematopoietic stem cells also leads to an upregulation of interferon-stimulated genes (ISGs) (9). Consistently, recent results from several groups show that deletion of ADAR1 in mice causes an upregulation of ISGs (10,11). The knock-out phenotype can partly be rescued by deletion of MDA5 or MAVS, which are part of the interferon response pathway of innate immunity (10,11). Furthermore, mutations in the *ADAR1* gene cause Aicardi-Goutières syndrome, which is a fatal autoimmune disease in children caused by an upregulation of ISGs (12).

Recent reports also show that ADAR1 and ADAR2 are aberrantly expressed in several cancers (reviewed in (13)). The ADAR1 gene is frequently amplified in cancer cells resulting in increased editing activity (14,15). In contrast, reduced ADAR2 editing activity without a clear effect on ADAR2 mRNA expression has been reported in glioblastoma (16). Overexpression of ADAR1 in both cell culture and mouse models contributes to the malignant phenotype and acts as a driver of development of cancer hallmarks such as cell proliferation, migration and invasion (17–20). In alignment, ADAR1 silencing in breast cancer cell lines leads to a significant increase in apoptosis, suggesting that ADAR1 may act as an anti-apoptotic factor and promote cancer progression (21). Therefore, the elevated levels of ADAR1 in different types of cancer presents a therapeutic opportunity to inhibit ADAR1, and thereby induce an innate immune response and cell death, specific to cancer cells. Yet, presently high-throughput screening for inhibitory compounds of ADAR1 is limited by the lack of a suitable reporter that quantitatively monitors editing activity in mammalian cells.

Here, we present a highly sensitive and quantitative bioluminescent reporter that enables variations in editing activity to be monitored in high-throughput setups. Stably introduced in the genome of two cancer cell lines, MCF7 and HeLa, the reporter enables detection of elevated editing activity due to an activation of ADAR1^p150^ by interferon-alpha as well as reduced editing activity due to ADAR1 or ADAR2 silencing. A pilot screen of a 33 000 compound chemical library demonstrates that cell lines carrying the reporter selectively identify modifiers of editing in high-throughput setups.

## MATERIALS AND METHODS

### Plasmids

**Figure 1.**
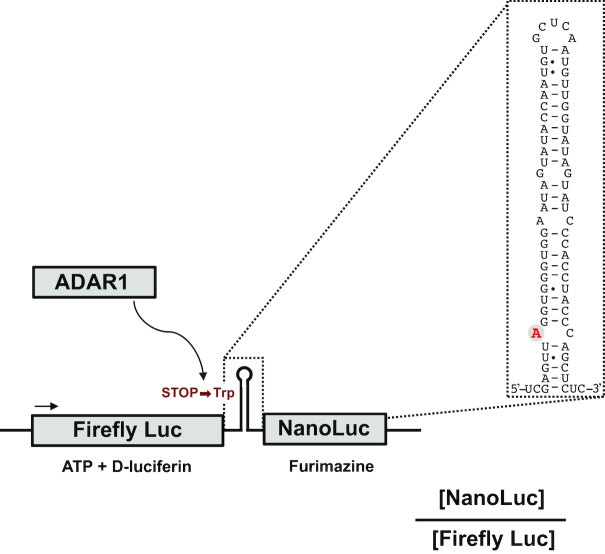
Dual luciferase reporter monitoring A-to-I editing. The reporter utilizes the natural GluA2 editing substrate in which the R/G editing site has been modified into a stop codon (U**A**G) that upon editing is recoded into a tryptophan codon (U**G**G). A firefly luciferase reporter gene upstream of the edited site monitors translation and a nanoluciferase reporter gene downstream is used to measure read-through after editing. Editing is measured as the ratio between luminescence from nanoluciferase and firefly luciferase.

The sequence including the R/G editing site from GluA2 (Figure 1), was synthetically made with homologous ends to the target vector (Integrated DNA Technologies). The Nanoluciferase (Nluc) sequence was polymerase chain reaction (PCR)-amplified from the NlucPEST yeast expression plasmid that has previously been described (22). The R/G editing and Nluc sequences were inserted by homologous recombination in yeast into vector pCA923 (23), after the firefly luciferase gene under a pTDH3 promoter. A plasmid for transient expression of the reporter in mammalian cell culture was created by replacing the yeast pTDH3 promoter with a PCR-amplified mammalian CMV promoter by homologous recombination in yeast. A positive control of the reporter was generated by site-directed mutagenesis (QuikChange II XL Site-Directed Mutagenesis Kit, Agilent Technologies) of the editing site to a G, mimicking permanent editing. Similarly, a negative control was obtained by deletion of 18 nt in the editing complementary sequence which is required for editing.

The Lenti plasmid for generation of cell clones stably expressing the reporter was made by PCR-amplification of the reporter from the plasmid described above with overhangs to a pLenti-puro plasmid (Addgene #39481). The PCR product and the pLenti vector were assembled using NEBuilder HiFi DNA Assembly (New England Biolabs) according to the manufacturer's instructions.

The ADAR1 mammalian expression vector pCS DRADA-FLIS6 (24) was a kind gift from Mary O’Connell. The ADAR2 expression vector has been previously described (25,26).

The ADAR1 yeast expression vector was constructed by PCR amplification of ADAR1 from the mammalian ADAR1 expression vector pCS DRADA-FLIS6 using primers with homology to target vector pLA1 (27) followed by homologous recombination in yeast.

### Yeast cell culture

Yeast cells were grown at 30°C in synthetic complete medium (SC) with 2% glucose to select for plasmids introduced by standard transformation procedures (28). To induce ADAR1 expression, overnight cultures were shifted to SCGal (2% galactose).

### Human cell culture

MCF7 was cultured in Dulbecco's modified eagle's medium (DMEM) (Life technologies) complemented with 4500 mg/l Glucose, 10% FBS (GE Healthcare), 4.5 mM L-Glutamine (Life technologies) and 100 U/ml penicillin/streptomycin. The Lenti-X 293T cell line was purchased from Clontech. HEK293, HeLa and Lenti-X 293T cells were cultured in DMEM supplemented with 10% FBS, 4500 mg/l Glucose, 4.5 mM L-Glutamine, 1 mM sodium pyruvate, 1 × non-essential amino acid and 100 U/ml penicillin/streptomycin. In the analyses, cells were cultured in media without penicillin/streptomycin. Cells were kept at 5% CO_2_ incubator at 37°C.

### Generation of stable cell clones

The Lenti plasmid containing the reporter was co-transfected with the lentiviral envelope plasmid pVSVg (Addgene #8454) and packaging construct psPAX2 (Addgene#12259) into Lenti-X 293T cell line. Packaged lentiviral particles expressing the reporter were harvested and transduced to either MCF7 or HeLa cells. Puromycin (Sigma-Aldrich) was added to the cells to a concentration of 1 μg/ml after 48 h, to select for positive cells. Single cell clones were selected with a cylinder (Sigma-Aldrich) after 10 days and passaged into a 96-well plate. Cell clones were expanded and then validated with reporter activity assay (described below). The RNA editing level of each clone was also determined by Sanger sequencing. The positive clone (+) was selected using the same procedure. Selected stable MCF7 and HeLa cell clones were cultured in the corresponding media containing 1 μg/ml puromycin.

### Transfection and siRNA knockdown

Cells were plated at 300 000 cells per well in duplicates in 12-well plates and after 24 h subjected to transfection. The editing reporter plasmids (including positive and negative controls) were transfected at 750 ng/well using Lipofectamine 2000 (Thermo Fisher Scientific) according to the manufacturer's instructions. ADAR1 or ADAR2 expression vectors were co-transfected at 0, 50, 250, 500, 750 and 1000 ng/well or 0, 10, 25, 50, 75 and 100 ng/well respectively. Empty vector (pcDNA3) was transfected to obtain an equal amount of DNA in each well. Forty-eight hours post-transfection, cells were harvested and the reporter activity was measured as described below. For ADAR knockdown, cells were plated at 100 000 cells per well in duplicates in 12-well plates. After 24 h, cells were transfected with either siADAR1 (siGENOME Human ADAR siRNA-SMARTpool, catalog number M-008630-01-0005, Dharmacon), siADAR2 (siGENOME human ADARB1 siRNA-SMARTpool, catalog number M-009263-01-0005, Dharmacon) or siCtrl (siGENOME non-targeting siRNA pool, catalog number D-001206-13-05, Dharmacon) at 25 pmol per well by Lipofectamine 2000 (Thermo Fisher Scientific) according to the manufacturer's instructions. Cells were harvested after 48 h and the reporter activity was measured as explained below.

### Induction of ADAR1 by IFN-α

HeLa-Nluc-edit and MCF7-Nluc-edit cells were plated at 25 000 cells/well in a Nunc F96 Microwell white plate (Thermo Fisher Scientific). IFN-αA/D (I4401, Sigma-Aldrich) was added to the medium at point of plating at concentrations 0, 10, 25, 50, 100 and 200 U/ml. After 24 h, the reporter activity was measured directly in the plate as explained below.

### Reporter activity assay

Yeast cells (100 μl) were transferred directly from the culture to a Nunc F96 Microwell white plate (Thermo Fisher Scientific). Bioluminescence was detected in an Orion II Microplate Luminometer (Berthold Technologies) as described previously (22,23). Briefly, firefly luciferase bioluminescence was measured after addition of 50 μl of 455 ng/μl D-luciferin (GoldBio) using the injection system of the instrument. For Nanoluciferase bioluminescence determination, 3 μl nanoluciferase substrate from Nano-Glo Luciferase Assay System (Promega) was added manually in a separate well containing 30 μl yeast culture from the same sample. For transiently transfected human cells and ADAR knockdown experiments in reporter cell clones, 250 μl 1 × Passive Lysis Buffer (Dual Luciferase Reporter assay system kit, Promega) was added to each well and plates were incubated for 15 min on a rotator at room temperature. A total of 20 μl of the supernatants was transferred to a Nunc F96 Microwell white plate (Thermo Fisher Scientific) and 20 μl ONE-Glo Luciferase Assay System reagent (Promega) or, alternatively, Nano-Glo Luciferase Assay System reagent (Promega) was added to the duplicates, respectively. Bioluminescence was detected using the Orion II Microplate Luminometer. For the interferon experiments, medium was removed from each well in the Nunc F96 Microwell white plate and 10 μl of ONE-Glo Luciferase Assay System reagent or Nano-Glo Luciferase Assay System reagent was added in separate wells and luminescence was detected using the same instrument as above. Cell lysis reagent is included in the substrate products.

### Western blotting

Total yeast protein samples were prepared as described previously (29), by mixing 1 ml yeast culture with 250 μl 1.85 M NaOH and incubated 5 min on ice. Thereafter, 250 μl 50% trichloroacetic acid was added and cells were pelleted and resuspended in 100 μl 1 M Tris base. Cells were then pelleted again and resuspended in 2× NuPAGE Sample Buffer, 4% sodium dodecyl sulphate with reducer (10 μl per 0.2 OD_600_ and ml culture). The OD_600_-normalized samples were boiled for 5 min and 15 μl per sample was loaded on NuPAGE precast gel and run at 120 V for 20 min and then 150 V for 1 h. Proteins were transferred at 120 V for 1 h and the membrane was then blocked for 1 h in 5% milk. Primary antibodies, anti-FLAG (Sigma #F7425; 1:1000) and anti-PGK1 (Life Technologies; 1:10 000) were diluted in 5% milk and incubated for 1 h with the membrane. Blots were washed with Tris-buffered saline (TBS) containing 0.05% tween and incubated with corresponding secondary antibodies, washed and analyzed with an Odyssey FC Imager (LI-COR Biosciences).

For detection of protein expression in human cells, cells were lysed in complete lysis-M, EDTA-free buffer (Roche) with 1% Protease Inhibitor cocktail (Roche), 1 mM PMSF, 1% PhosphoStop (Roche). Cells in lysis buffer were incubated for 10 min at 4°C and then centrifuged for 15 min at 14 000 *g*. Supernatants were transferred to a new tube and protein concentration was measured using Bradford assay. Equal amounts of total protein were mixed with 2 × Laemmli sample buffer (Bio-Rad) containing 5% β-mercaptoethanol (Sigma) and boiled for 10 min. After cooling on ice, denatured proteins were loaded to 10% gradient mini-protein TGX precast 10 well gels (Bio-Rad) and run for 1 h at 120 V. Proteins were then transferred to PVDF membranes for 1 h and 15 min at 120 V. After blocking with 5% bovine serum albumin for at least 1 h, membranes were blocked with primary antibodies overnight at 4°C. Primary antibody anti-ADAR1 (sc-73408, Santa Cruz, 1:500), anti-ADAR2 (#HPA018277, Sigma-Aldrich, 1:250), anti-Firefly Luciferase (ab21176, Abcam, 1:1000) and anti-β-actin (AC-15, A1978, Sigma-Aldrich, 1:20 000) were used. Blots were washed with TBS containing 0.1% tween and incubated with corresponding Horseradish Peroxidase (HRP)-conjugated secondary antibodies (DakoCytomation), washed and developed using WesternBright Sirius detection kit (Advansta) and ChemiDoc XRS+ camera.

### RNA extraction, real time-PCR and Sanger sequencing

RNA was isolated from human cell lines using GenElute™ Mammalian Total RNA Miniprep Kit (Sigma-Aldrich) and from yeast using a RiboPure RNA Purification Kit for Yeast (Ambion). To remove possible DNA contamination, total RNA was treated with DNase I (Thermo Fisher Scientific). A total of 1 μg DNase-treated RNA was reverse transcribed using random hexamers (Invitrogen) and SuperScript II (Thermo Fisher Scientific) according to the manufacturer's protocol. To ensure absence of DNA contamination, cDNA synthesis without SuperScript II was performed for each sample. To determine RNA editing at the stop codon in the reporter, RT-PCR was performed with forward primer: 5′-CATTTGATCACCAGATAAACC-3′ and reverse primer: 5′-CTTACCGGAAAAGTCGACGC-3′ ordered from Eurofins Genomics. Each PCR reaction consisted of 1 × PCR Buffer (-MgCl) (Thermo Fisher Scientific), 1.5 mM MgCl, 0.2 mM dNTP mix, 0.2 μM forward and reverse primers, 0.5 μl of Taq DNA polymerase (5 U/μl, Thermo Fisher Scientific) and 2 μl cDNA in a total volume of 50 μl. The PCR reaction started with denaturing for 4 min at 95°C, followed by 35 cycles with 20 s at 95°C, 20 s at 55°C and 30 s at 72°C and then 7 min at 72°C. PCR products were loaded on a 1% agarose gel and run for 50 min at 110 V. Purified PCR products were sent for Sanger sequencing at Eurofins Genomics. The percentage of editing was calculated from the peak height of G/(A+G) × 100.

### High-throughput screening assay

HeLa-Nluc-edit cells were seeded at a density of 15 000 cells per well into 384 white plates (PerkinElmer, CulturPlate-384, #6007689) in 20 μl of Opti-MEM without phenol red (Thermo Fisher Scientific #11058021) using a multidrop dispenser (Thermo Fisher Scientific) and incubated overnight at 37°C, 5% CO_2_. The cells were then treated with compounds (see below) for 24 h prior to luminescence signal detection. Briefly, 10 μl of Nluc GLOW Assay reagents (Nanolight Technology # 325) were added and luminescence signal was measured within 10–20 min after the addition using a Victor 2 (Perkin Elmer) multiplate reader at 1 s integration time. Subsequently, 20 μl Fluc Assay reagents (Nanolight Technology # 318) were added to the same wells and luminescence signal was measured with Envision (PerkinElmer) multiplate reader at 0.1 s integration time, within 5–10 min after reagent addition. The ratio between Nluc and FFL signal was calculated for each well and results for each plate were normalized to a negative (0.1% DMSO) and positive (10 μM Nluc inhibitor 1) control. The assay quality was assessed by Z’ factor using the following formula: Z′-factor  =  1 − [(3(σp + σn)/(|μp − μn|))], where μn and σn represent the mean and standard deviation of the negative control, and μp and σp represent the mean and standard deviation of the positive control (30).

### Compound screening

A set of 33 000 compounds from the primary screening set at Science for Life Laboratory Compound Collection (https://www.scilifelab.se/wp-content/uploads/2017/08/scilifelab-compound-collection.pdf) was applied in the campaign. For screening purpose, the compound solutions (10 mM in DMSO) were transferred from Labcyte 384 LDVplates (LP-0200) to 384 polypropylene plates (Greiner # 781201) using an Echo 555 acoustic liquid handler (LabCyte). The compound solutions (60 nl) were dispensed into columns 1–22 and an equivalent volume of DMSO for the negative control (0% inhibition), and a 10 mM DMSO solution containing Nluc Inhibitor 1 (100% inhibition) for the positive control in columns 23 and 24, respectively. Plated compounds were stored at −20°C. The day of the experiment, the plates were thawed and 20 μl of OptiMEM were added to each well using a multidrop dispenser. A total of 10 μl of diluted compound solution were then transferred to the plates with cells growing in 20 μl medium, using Bravo liquid handling system (Agilent). The final compound concentration used in the screen was 10 μM, with a final DMSO concentration of 0.1% in all wells.

## RESULTS

### A dual bioluminescence reporter sensitive to small changes in RNA editing activity

We aimed at constructing a reporter to monitor editing activity in human cells that: (i) is sensitive to small fluctuations in editing activity, (ii) yields reproducible data points, (iii) has a quantitative readout suitable for high-throughput measurements and (iv) is sensitive to endogenous editing elicited by external stimuli or inhibitors. Although a number of excellent editing reporters have been described previously for the use in several eukaryotic organisms (31–35), none of them was suitable for high-throughput screening in human cells. Inspired by these reporters, we used the R/G editing site of the GluA2 transcript as a base for our construct (Figure 1) (31,33,36). The stem loop sequence was modified to introduce an amber stop codon (U**A**G) that upon editing (U**I**G) is read as a tryptophan. A firefly luciferase (FFL) gene expressed independently of the editing levels was introduced upstream of the editing site to enable internal normalization. A gene encoding Nanoluciferase (Nluc) was placed downstream of the stop codon editing site but in frame with FFL, only allowing single cistronic expression after editing. Thus, the Nluc/FFL bioluminescence ratio becomes a measure of editing activity in the cells.

We initially constructed and tested the proof-of-principle of the reporter in budding yeast (*Saccharomyces cerevisiae*) that is completely devoid of endogenous ADAR activity. The assembled reporter plasmid and an expression plasmid encoding FLAG-tagged human ADAR1 under the inducible *GAL1* promoter was introduced in yeast cells by transformation. Bioluminescence specific for FFL and Nluc was followed for 8 h after induction of ADAR1 expression by transfer to galactose medium (22). The editing activity was gradually increased and plateaued 6 h after transfer to inducing medium (Supplementary Figure S1A). Western analysis showed that the activity-increase was closely matched with ADAR1 protein levels (Supplementary Figure S1B). RNA editing of the reporter transcript was verified by sequencing after RT-PCR (Supplementary Figure S1C). Importantly, detection of RNA editing of the reporter transcript by Sanger sequencing revealed that the reporter was highly sensitive (Supplementary Figure S1C); despite modest editing of the accumulated mRNA, we obtained robust bioluminescent signals. For direct comparison, we mutated the U**A**G stop codon editing target into a U**G**G tryptophan codon, causing full read-through into the Nluc open reading frame. This resulted in 1000-fold increased reporter signal, emphasizing the sensitivity and detection range offered by the reporter (Supplementary Figure S1C).

### ADAR activity monitored in human cells

**Figure 2.**
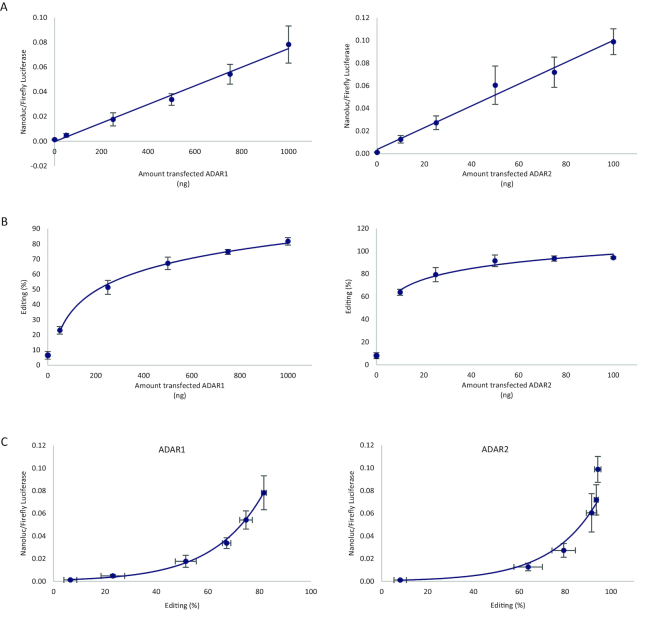
Highly detectable signal of editing in the reporter when transfected into HEK293 cells with cotransfected ADAR1 or ADAR2. Editing was determined as the ratio between luminescence from nanoluciferase and firefly luciferase (Nluc/FFL) expressed from the reporter plasmid. (**A**) Nluc to FFL signal ratio increased in a linear fashion when the reporter was cotransfected with increasing amounts of ADAR1 (left, *R*^2^ = 0.993, *n* = 3) and ADAR2 (right, *R*^2^ = 0.986, *n* = 3) expression vector. Error bars represent standard deviation. (**B**) Editing was verified by Sanger sequencing, and editing levels (G/(A+G) × 100) correlated logarithmically with the amount of cotransfected ADAR1 (left, *R*^2^ = 0.997, *n* = 3) and ADAR2 (right, *R*^2^ = 0.959, *n* = 3). Error bars represent standard deviation. (**C**) Reporter signal as a function of editing levels indicating that the Nluc/FFL ratio was exponentially dependent on editing percentage when the reporter was edited either by ADAR1 (left, *R*^2^ = 0.991, *n* = 3) or by ADAR2 (right, *R*^2^ = 0.983, *n* = 3). Error bars represent standard deviation.

We adapted the dual editing reporter construct for use in mammalian cells and tested it in HEK293 cells that are well suited due to low endogenous ADAR activity. Following cotransfection of ADAR1 and ADAR2 expression vectors with the reporter, the bioluminescent signal increased in a linear manner with the amount of applied plasmid DNA, indicating that the reporter transcripts were efficiently edited by either of the editing enzymes (Figure 2A). Moreover, the increase in reporter signal was in proportion to the R/G editing activity determined by Sanger sequencing of the reporter transcript after RT-PCR (Figure 2B). At high amounts of transfected ADAR1 or ADAR2 expression vector, the majority of the reporter transcripts were edited at the R/G site. While the reporter signal increased linearly with the amount of transfected ADAR1 or ADAR2 plasmid DNA, the increase in editing determined by Sanger sequencing displayed a logarithmic relationship (Figure 2B). Consequently, the luciferase reporter signal showed an exponential increase when plotted against editing percentage (Figure 2C). Expression of FFL alone (unedited) and as part of the fusion with Nluc was confirmed by western blot analysis of ADAR1-transfected HEK293 cells (Supplementary Figure S2A). Notably, the level of the fusion protein was very low compared to non-fused FFL despite robust editing (∼80%), suggesting that the fusion protein was more rapidly turned over than the unfused FFL. Western blot of a construct where the edited stop codon had been replaced by a tryptophan codon similarly showed lower levels of fusion protein compared to unfused FFL (Supplementary Figure S2B). A potential issue with editing reporters is that ADAR enzymes frequently edit additional sites in the vicinity of the specific adenosine target, which possibly could affect reporter activity. Yet we did not detect extensive off-target editing and only when ADAR1 was cotransfected at the highest amount (1000 ng) 12% editing of a site located 39 nucleotides downstream of the R/G site was found (data not shown). Thus, the dual luciferase construct faithfully reports on editing in HEK293 cells.

### Monitoring endogenous editing in cancer cells

**Figure 3.**
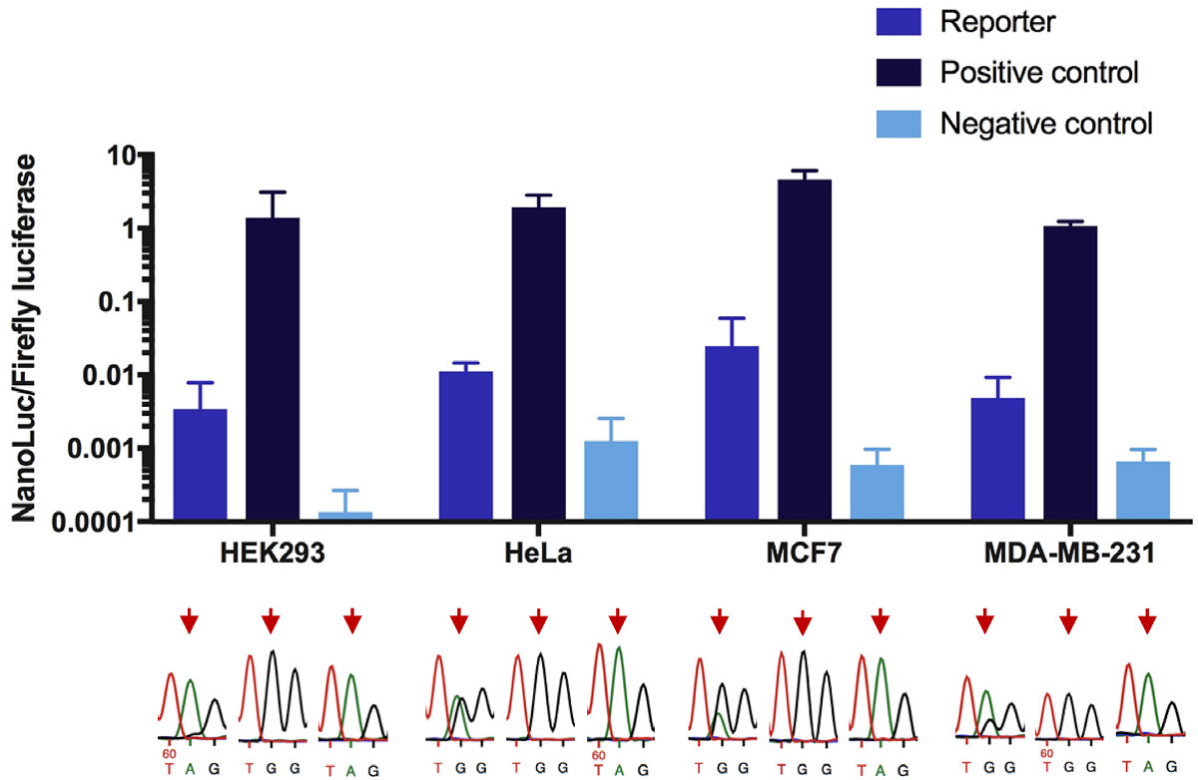
The transfected reporter sensing endogenous editing in different human cancer cell lines. Editing was determined as the ratio between luminescence from nanoluciferase and firefly luciferase (Nluc/FFL) in: HEK293, transformed human embryonic kidney cells; HeLa, cervical cancer cells; MCF7, breast cancer cells and MDA-MB-231, breast cancer cells. Positive control: the edited site (U**A**G) was mutated to Trp (U**G**G), mimicking 100% editing. Negative control: deletion of the editing complementary sequence prevented editing and was used as a negative control. Below, editing verified by Sanger sequencing of total RNA after RT-PCR and shown as a dual A and G peak in the chromatogram. Arrows indicate site of editing. Error bars indicate standard deviation (*n* = 3).

To test the sensitivity of the reporter to endogenous editing activity in cancer cell lines, the dual luciferase editing reporter was transiently transfected into three human cancer cell lines: HeLa, MCF7 and MDA-MB-231. The MCF7 breast cancer cell line has previously been shown to exhibit elevated ADAR1 expression and the frequency of editing is significantly higher in breast tumors compared to normal breast tissues (37). The HEK293 cell line was used as a control for low levels of endogenous editing activity. To explore the dynamic range of the reporter signal, two reporter controls were employed: (i) a positive control where the edited stop codon (U**A**G) was mutated to a Trp codon (U**G**G), functioning as a proxy for 100% editing and (ii) a negative control where the editing complementary sequence in the R/G stem had been removed, disrupting the double-strand at the site and thus preventing editing. A clear signal of Nluc, higher than the negative control, was seen in all tested cell lines, demonstrating the reporter's ability to detect endogenous editing (Figure 3). Chromatograms from Sanger sequencing of the corresponding cDNA showed variable levels of endogenous editing of the substrate in the different cell lines. The metastasizing MDA-MB-231 cell line showed a moderate level of reporter-editing, while the endogenous editing activity was high in both HeLa and MCF7 cells. Editing in HEK293 cells was as expected very low, but still reproducibly detectable by the reporter, indicating high sensitivity. The Nluc/FFL ratio measured from the positive control was extremely high in all cell lines, showing the broad dynamic range of the reporter.

### Stable expression of the editing reporter in different cancer cell lines

**Figure 4.**
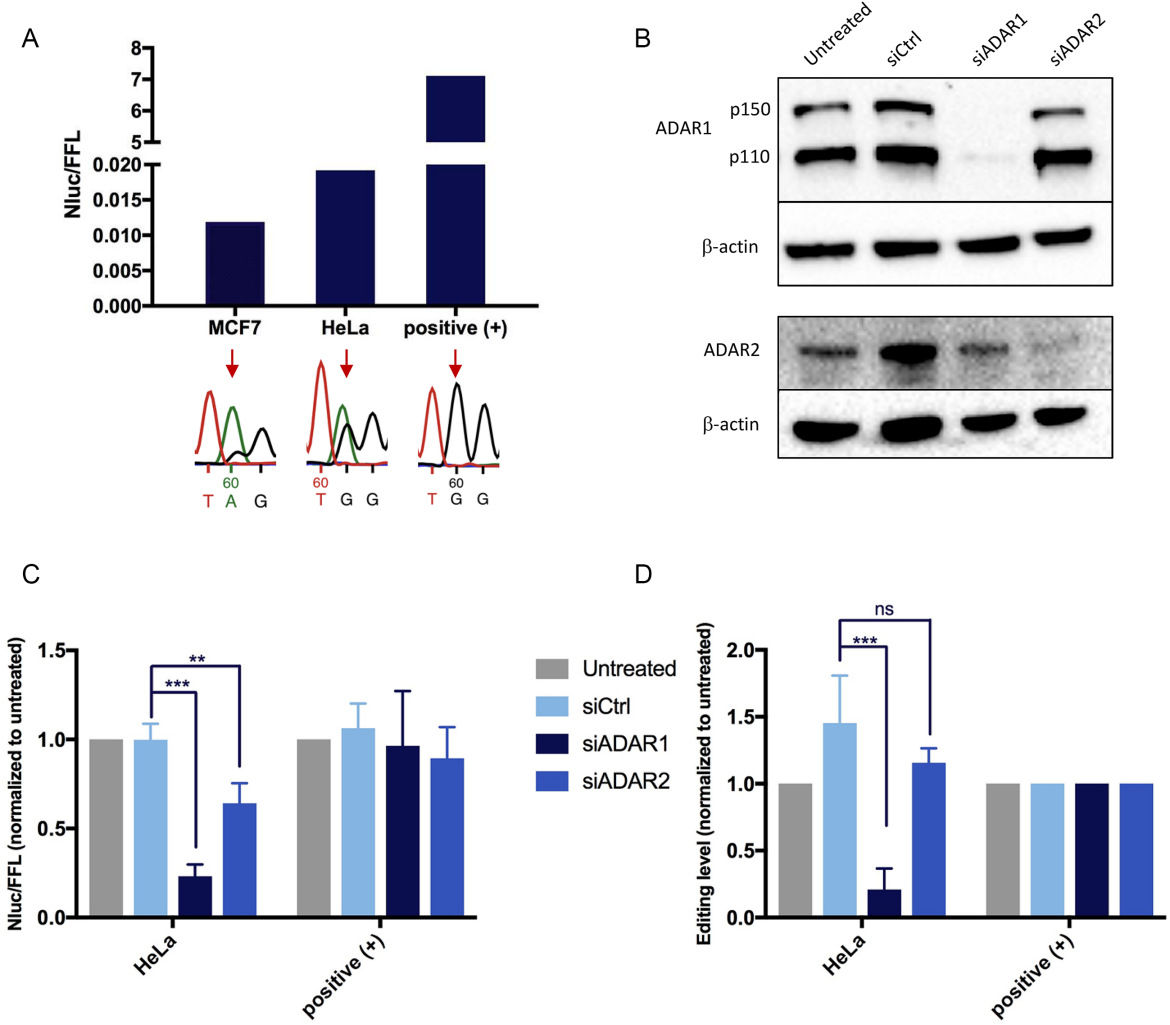
Analysis of endogenous editing in cancer cells stably expressing the editing reporter. (**A**) Editing, monitored as the Nluc/FFL ratio, of the reporter and a positive control with a permanent G at the editing site, stably expressed in MCF7-Nluc-edit and HeLa-Nluc-edit cells. Editing was verified by Sanger sequencing, seen as a dual A/G peak in the chromatogram (red arrow). (**B**) ADAR1 and ADAR2 knockdown efficiency in HeLa-Nluc-edit cells, demonstrated by representative western blots. β-actin was used as loading control. (**C**) Reporter activity of HeLa-Nluc-edit cells transiently transfected with siADAR1, siADAR2 or scramble siCtrl (*n* = 4) and normalized to untreated. (**D**) Relative RNA editing levels of reporter clones transiently transfected with siADAR1, siADAR2 or scramble siCtrl (*n* = 4) and measured by Sanger sequencing after RT-PCR. RNA editing levels were determined by measuring the peak heights in chromatograms after Sanger sequencing (G/(A+G) × 100). As expected the positive control was unaffected by ADAR knockdown. Error bars indicate standard deviation. Significance testing was performed using two sides Student's *t*-test (** indicates *P* < 0.01; *** indicates *P* < 0.001 and NS, not significant).

To permit high-throughput screening of modifiers of editing activity, we generated stable MCF7 and HeLa cell clones containing the reporter. Following lentiviral transduction of the reporter, the selected MCF7 and HeLa clones, hereafter referred to as MCF7-Nluc-edit and HeLa-Nluc-edit, were edited to a level of 15 and 39%, respectively (Figure 4A). A stable HeLa clonal line with the positive control containing a G at the editing site of the reporter was also generated. To determine the specificity and efficiency of the editing reporter, ADAR1 and ADAR2 were knocked down by specific siRNAs. The efficiency of the respective knockdown was demonstrated by Western blotting (Figure 4B). The editing activity measured by the reporter was reduced after treatment of siRNA against both ADAR1 and ADAR2, compared to either the untreated or siCtrl, but more prominently by siADAR1 (Figure 4C). As expected, editing activity was not changed in the positive control. Editing of the reporter was verified by Sanger sequencing and shown to be greatly decreased after ADAR1 silencing, in line with the result from the reporter assay (Figure 4D). These results show that ADAR1 plays a major role in editing of the reporter RNA hairpin in HeLa cells and is consistent with previous evidence pointing to high ADAR1 activity in cancer cells (17–20).

### Faithful detection of external editing modifiers

**Figure 5.**
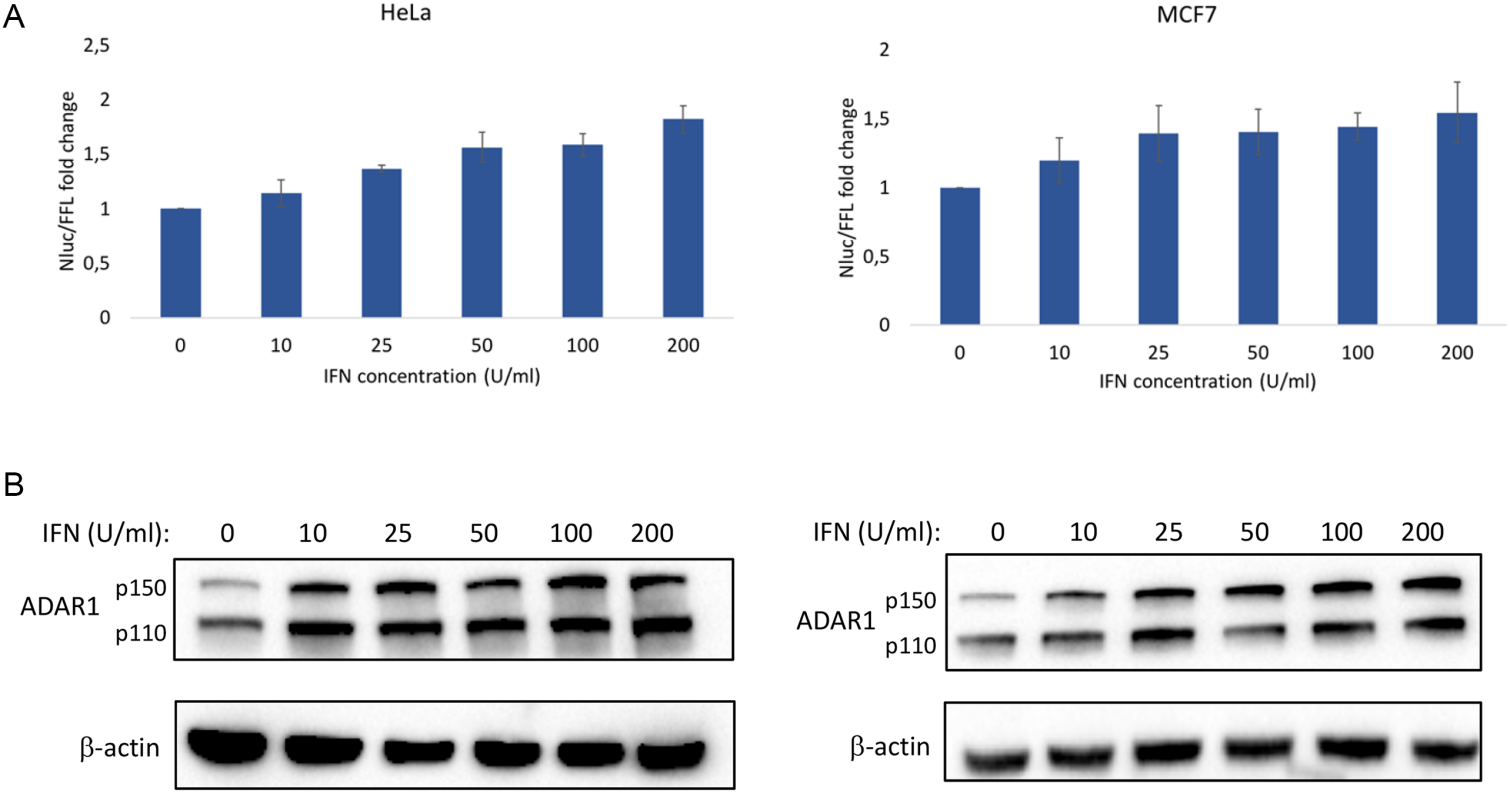
Induction of A-to-I editing by IFN-α in HeLa-Nluc-edit (left) and MCF7-Nluc-edit (right) cells stably expressing the reporter. (**A**) The reporter signal, representing editing, increased gradually by up to 1.8-fold in HeLa and 1.5-fold in MCF7 at 200 U/ml IFN-α compared to the untreated controls. Error bars indicate standard deviation (*n* = 4). (**B**) Western blots showing the induction of ADAR1 in the IFN-treated cells compared to untreated. The interferon-inducible long form of ADAR1 is indicated by p150 and the short form by p110. β-actin was used as loading control.

We directly tested if the HeLa-Nluc-edit and MCF7-Nluc-edit lines were useful to monitor fluctuations in endogenous ADAR1 expression upon exposure to external stimuli. The clonal lines were exposed to up to 200 U/ml interferon-alpha (IFN-α), an inducer of the expression of ADAR1^p150^ (8) and the reporter signal was determined after 24 h. IFN-α elevated editing in a dose-dependent manner by up to 1.8-fold in HeLa-Nluc-edit and 1.5-fold in MCF7-Nluc-edit at the highest concentration compared to the respective untreated controls (Figure 5A). Editing was confirmed by Sanger sequencing after RT-PCR of the reporter RNA (Supplementary Figure S3). Increasing the IFN-α levels beyond 200 U/ml did not lead to further increase in reporter signal (data not shown), suggesting that editing was maximally induced at this concentration. Western blotting detected a clear increase in ADAR1 ^p150^ levels upon IFN-α treatment, which was well correlated with the increase in editing activity (Figure 5B). These results demonstrate that the reporter is sensitive enough to detect subtle changes in endogenous editing activity.

### Development of a high-throughput screening platform

To develop an assay compatible with the high-throughput screening platform in 384-well plates, we selected the HeLa-Nluc-edit cell line due to its high-editing phenotype. To this end we simplified the luminescence detection by using single-well measurement of both luciferases rather than the previously used two-well method based on the Nano-Glo and ONE-Glo reagent. The commercially available Dual-Glo-Nano Promega kit allows for sequential measurement of FFL and Nluc level in the same well, however, we found that the Nluc signal was significantly lower than for Nano-Glo reagents (Supplementary Figure S4A). We overcame this problem by using custom made kits from NanoLight Technology. The signal obtained for Nluc detected with Nluc Assay reagents was almost 2-fold higher than when using Nano-Glo kit from Promega (Supplementary Figure S4B). In addition, higher dynamic range of Nluc/FFL signal ratio allowed us to increase the number of cells to up to 15 000 per well and thereby obtain a robust Nluc signal. Nluc signal was stable and did not change significantly for up to 1 h after addition of the reagents, whereas the half-life for FFL signal was 1 h (data not shown).

We used Nluc inhibitors as positive controls for assessing the reproducibility and robustness of the screening assay. Two Nluc inhibitors were synthesized according to previously published method (38) and tested in our assay set-up. Both inhibitors were significantly decreasing Nluc signal in a concentration-depended manner, without alterations of FFL signal. IC50 values were determined based on the ratio between Nluc and FFL signal and were found to be 0.08 and 0.06 μM, respectively, for inhibitor 1 and 2 (Supplementary Figure S5). Treatment with 10 μM of inhibitors for 24 h was then conducted to confirm reproducibility of the positive control response and used to derive a Z’ factor (0.1% DMSO was used as a negative control; vehicle). The ratio between Nluc and FFL signals showed excellent Z’ factor values (above 0.7) for both inhibitors when the Nluc signal was measured within 25 min and FFL signal within 5 min after reagent addition, respectively. Longer incubation time caused a decrease of the Z’ factor. However, acceptable values around 0.4 were obtained up to 2 h after reagent addition (Supplementary Table S1) and no significant plate effects were observed (Supplementary Figure S6A). Up to 3% DMSO concentration did not change Nluc/FFL signal ratio, although higher signals were observed at lower concentrations; 0.5% for Nluc and 1% for FFL respectively (Supplementary Figure S6B).

### Screening of a small molecule library

**Figure 6.**
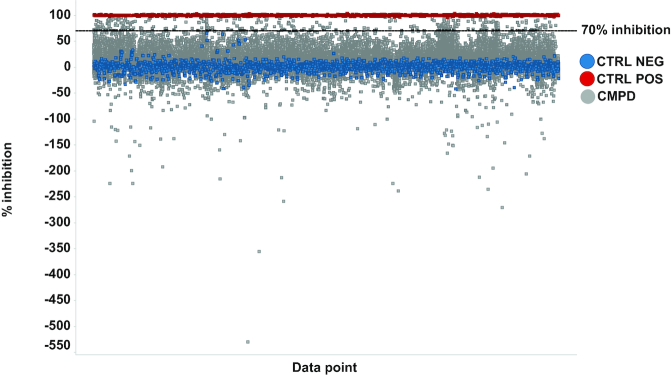
Illustration of the results from screening of a diverse library of 33 000 small molecules in HeLa-Nluc-edit cells. Percentage inhibition (Nluc/FFL signal) of tested wells grouped by plates, after filtering out substances causing inhibition of FFL signal. Wells treated with 0.1% DMSO (CTRL NEG 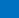), 10 μM Nluc Inhibitor 1 (CTRL POS 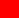) and 10 μM compound (CMPD 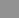). The dashed line represents the hit threshold (70% inhibition) applied for hit identification.

As a proof-of-principle for the functionality of the reporter, we used the optimized 384-well single well assay and screened a diverse library of 33 000 small molecules from the SciLifeLab compound collection (see: https://www.scilifelab.se/wp-content/uploads/2017/08/scilifelab-compound-collection.pdf) at a single concentration of 10 μM. The screening performance was very good as assessed by the Z’ factor calculated for cells treated with 10 μM Nluc inhibitor 1 (positive control) and 0.1% DMSO vehicle (negative control) and gained an average value of 0.67 ± 0.11 over all screening plates. To allow comparison of results from different plates and screening runs, all the results were normalized to the positive (100% inhibition) and negative control (0% inhibition). Percent inhibition values were calculated based on the ratio between Nluc and FFL signals and were used as a readout to select active compounds. In parallel, FFL signal was monitored and compounds causing higher than 40% inhibition of FFL signal were filtered out since they likely were toxic and may act as unspecific reporter inhibitors. Finally, with a threshold set to 70% inhibition, 300 compounds were selected as active, giving a primary hit rate of 0.9%. Interestingly, also small molecules significantly increasing Nluc/FFL signal were identified indicating that the reporter system facilitates the identification of both activators and inhibitors of editing (Figure 6). The primary hits were retested in independent experiments at 2.5, 5 and 10 μM. With some exceptions, most compounds reproducibly inhibited editing yielding values comparable to what was obtained in the primary screen (Supplementary Figure S7). This demonstrates that the assay is robust and has applicability in high-throughput screening of small molecule libraries.

## DISCUSSION

We have developed a reporter assay that can be used to monitor endogenous editing activity in mammalian cells. The assay is designed for high-throughput screening of inhibitors and inducers of ADAR1 and ADAR2 editing activity. We show that the reporter is highly sensitive and suitable to screen a large library of small molecules. The reporter signal is stable in the absence of compound (negative control) and responds well to positive controls consisting of Nluc inhibitors. Both inhibitors and activators of editing were identified in our primary screen. Hits from the primary screen could then be validated in a second step with three different compound doses, demonstrating the robustness of the assay. Follow-up experiments including counter-screening will have to be performed to verify the compounds as editing modifiers and rule out any false positive hits that could come from compounds that affect either of the luciferases but this is beyond the scope of this report. The modified GluA2 editing substrate is recognized by both ADAR1 and ADAR2, making the reporter very versatile and useful for a multitude of purposes. Nevertheless, the enzyme specificity of editing modifiers will have to be further investigated in downstream applications.

Next generation sequencing and The Cancer Genome Atlas (TCGA) have detected a substantial number of alterations in A-to-I editing in many different cancer types (37,14,15). Commonly ADAR1 levels are elevated resulting in an increased editing activity in many different types of cancers, whereas ADAR2 activity often is downregulated, particularly in brain tumors (reviewed in (13)). Chemical compounds that inhibit ADAR1 or enhance ADAR2 activity could therefore have potential as anti-cancer drugs. Currently, only a few inhibitors and enhancers of A-to-I RNA editing have been described (8,39,40). With the here presented reporter system, we pave the way for high-throughput screening with the aim of finding editing modifiers with the potential of clinical use.

Accurate levels of ADAR1 and ADAR2 are important for health, and aberrant editing not only occurs in cancer but also in other diseases. Aicardi-Goutière's syndrome (AGS) is a disorder that manifests in infants and young children and is caused by mutations in ADAR1 (12). Symptoms mimic congenital infection with upregulation of IFN and IFN-stimulated genes (41). The disease profile suggests reduced activity of the editing enzyme since the symptoms are reminiscent of ADAR1 deficiency in mouse (10,11). Boosting ADAR1 activity using an editing inducing compound therefore has potential in the treatment of this disease, especially since editing activity in AGS is reduced globally due to genetic mutations in ADAR1, and treatment that induces ADAR1 activity therefore is less likely to have dangerous adverse effects.

The Nluc/FFL ratio from our reporter was found to be an excellent tool to determine editing activity, even though the luminescence ratio is lower than we first expected as it has previously been shown that the luminescence emitted in the Nluc enzymatic reaction is more than 150-fold stronger than that of the FFL enzyme (42). This would in our system mean that an editing level of 1% would yield a Nluc/FFL ratio of >1.5, and 100% editing would give a ratio of >150. We believe this deviation from the expected results to be a consequence of a difference in protein or bioluminescent stability between the FFL enzyme alone (produced from unedited transcripts) and the FFL-Nluc fusion (produced from edited transcripts). We base this conclusion on the fact that FFL expression levels are much higher than the levels of FFL-Nluc even at high editing levels (Supplementary Figure S2). We also consider that the variability of the Nluc/FFL ratio between cell types indicates that factors other than editing play a role for final luciferase activities, and that comparisons between cell types should be made with caution. For example, the FFL-Nluc fusion protein may be recognized by the cellular protein quality control machinery that targets aberrant proteins for degradation. We have also previously documented that N-terminal fusion to Nluc triggers activity loss and degradation of the luciferase (22).

The here described bioluminescent ADAR activity reporter offers cost-effective detection with high sensitivity, dynamic range and reproducibility, making the system highly suitable for high-throughput screening for editing modifiers. Although very useful for other purposes, earlier designs of ADAR editing reporters have mainly been based on yeast growth assays with a lower dynamic range and the modifier hits have been analyzed in low-throughput setups for quantitative determination of editing levels (33,36). However, a more recently developed editing reporter system in yeast was based on a more quantitative GFP reporter but excitation with blue light frequently limits sensitivity since yeast cells are highly autofluorescent (32). With our reporter, the result is quantitative also in the high-throughput format of human cells, eliminating the need for secondary reporter systems and outperforming western analysis and Sanger sequencing in speed and sensitivity.

## Supplementary Material

Supplementary Data
