# Supplementary material for: Sensitive ADAR editing reporter in cancer cells enables high-throughput screening of small molecule libraries
Source: Nucleic Acids Res. 2018 Dec 22;47(4):e22. doi: 10.1093/nar/gky1228 (PMC6393238; doi:10.1093/nar/gky1228)
Supplement: Supplementary Data [file gky1228_supplemental_files.pdf]

Table S1

Incubation with Nluc reagents	Incubation with FFL reagents	Inhibitor 1 [Z']	Inhibitor 2 [Z']
10 min	5 min	0.73	0.71
	30 min	0.69	0.67
	60 min	0.67	0.66
	120 min	0.59	0.58
25 min	5 min	0.73	0.74
	30 min	0.70	0.70
	60 min	0.67	0.68
	120 min	0.59	0.60
45 min	5 min	0.61	0.61
	30 min	0.56	0.57
	60 min	0.54	0.55
	120 min	0.48	0.49
90 min	5 min	0.48	0.50
	30 min	0.45	0.46
	60 min	0.44	0.45
	120 min	0.40	0.41

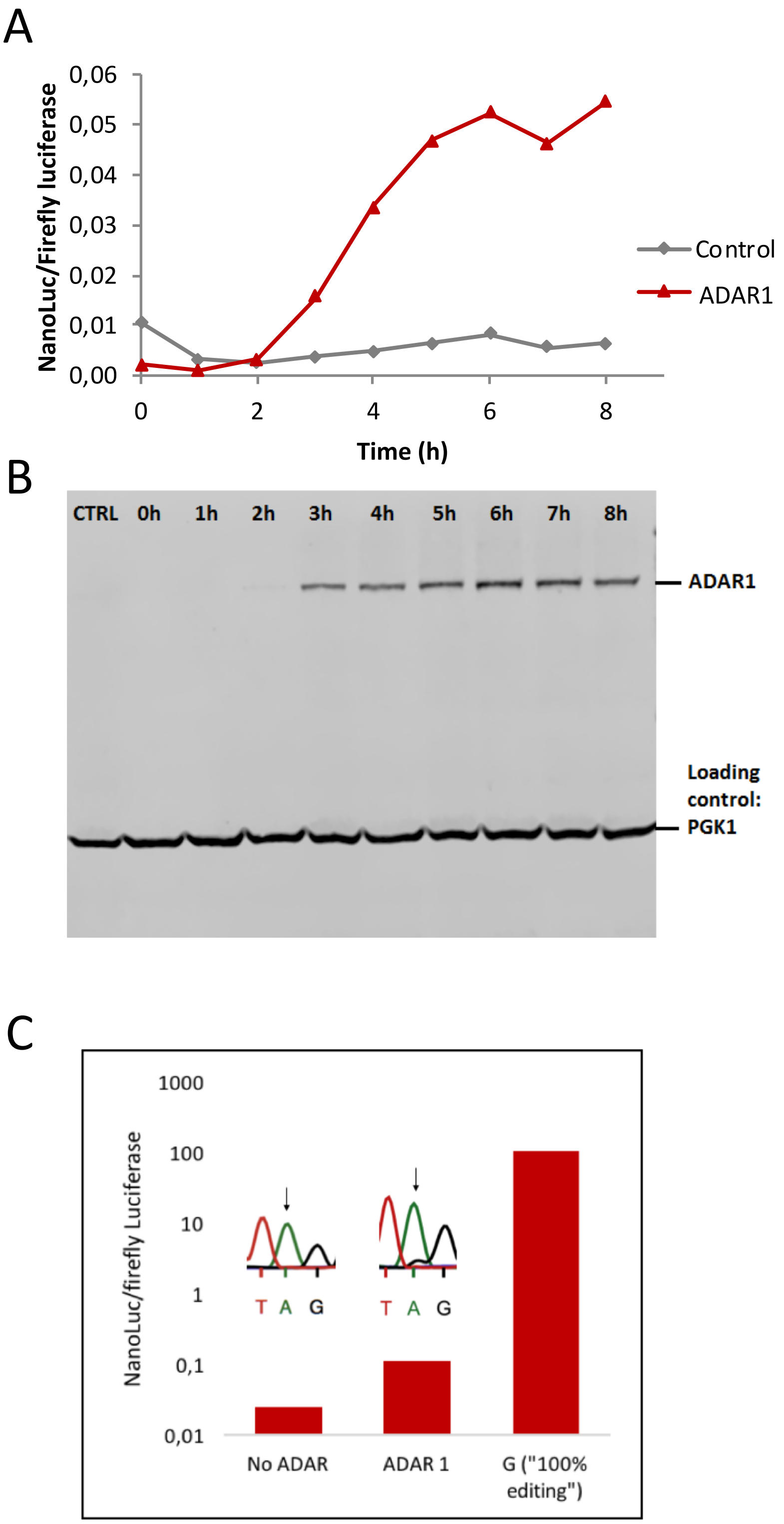

Supplementary Figure 1: A-to-I RNA editing reporter in yeast. (**A**) Signal of editing in the reporter in yeast expressing ADAR1 under the inducible GAL1-promoter at different time points after its induction. Editing was determined as the ratio between luminescence from nanoluciferase and firefly luciferase (Nluc/FFL) expressed from the reporter. In comparison, the reporter signal from a control strain lacking ADAR1 is shown. (**B**) Western blot of transiently expressed ADAR1 corresponding to each time point of reporter measurement. Yeast does not express ADAR endogenously. (**C**) The reporter signal with chromatograms from Sanger sequencing showing editing as a dual A/G peak. Editing is low (~10%), but gives a reproducible signal from the reporter. As a control a G was introduced at the editing site, mimicking 100% editing. Note that the scale is logarithmic.

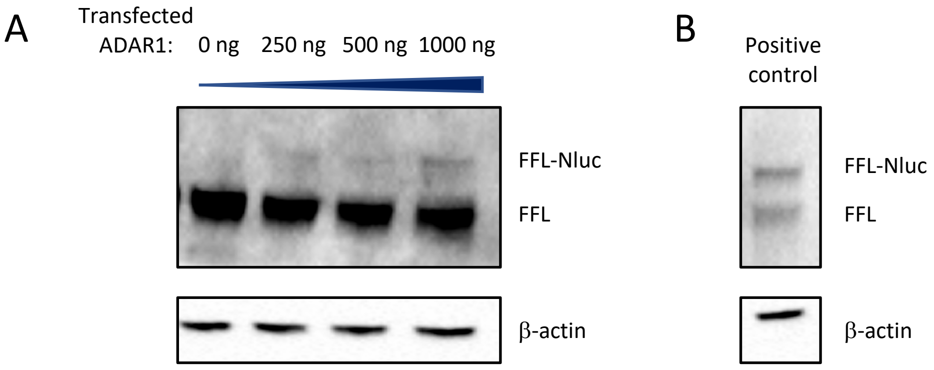

Supplementary Figure 2: Western blot showing Firefly luciferase (FFL) both as a single protein (translated from unedited transcripts) and as part of the fusion protein with Nanoluciferase (Nluc) (translated from edited transcripts) in A) Reporter-transfected HEK293 cells cotransfected with ADAR1 and B) HEK293 cells transfected with a reporter construct where the edited stop codon was mutated to a Trp codon (U**G**G) mimicking 100% editing (positive control). β-actin was used as a loading control.

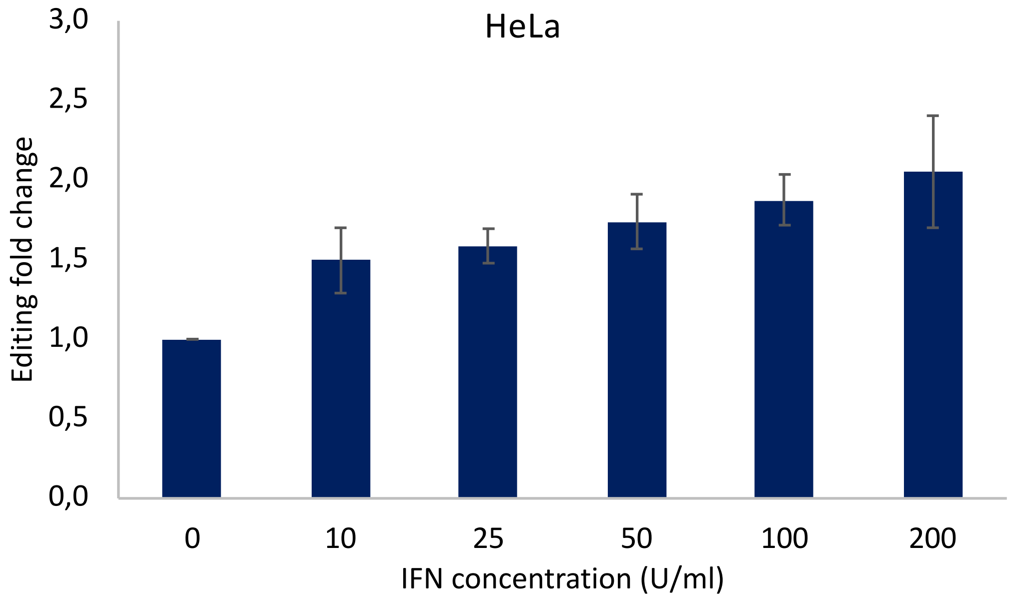

Supplementary Figure 3: Editing of the reporter as determined by Sanger sequencing after treatment of HeLa cells, stably expressing the reporter, with different concentrations of interferon-α (IFN). Editing levels were normalized to untreated. Error bars indicate standard deviation (n = 3).

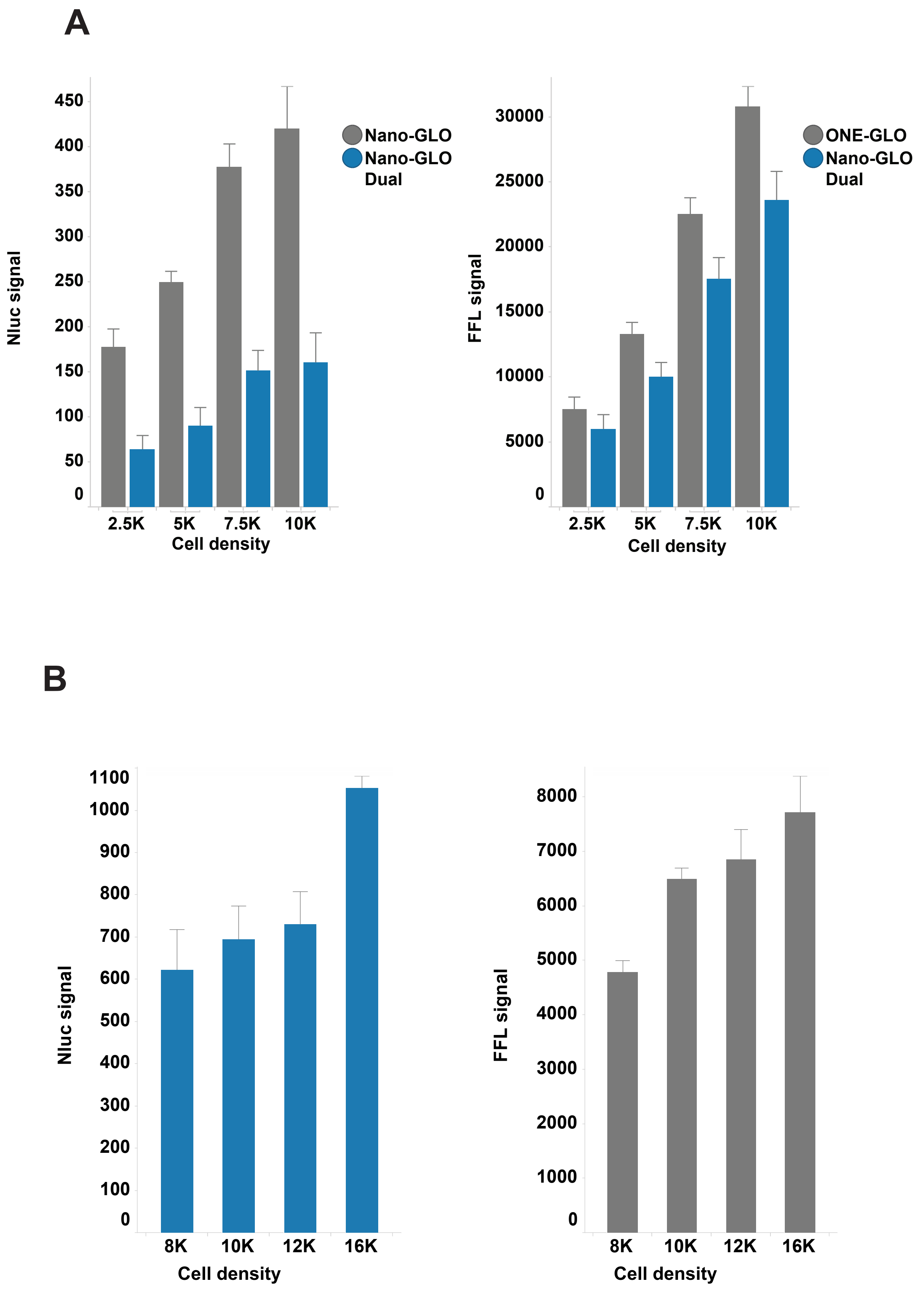
A
Nluc signal
450
400
350
300
250
200
150
100
50
0
Nano-GLO
Nano-GLO Dual
2.5K
5K
7.5K
10K
Cell density
FFL signal
30000
25000
20000
15000
10000
5000
0
ONE-GLO
Nano-GLO Dual
2.5K
5K
7.5K
10K
Cell density
B
Nluc signal
1100
1000
900
800
700
600
500
400
300
200
100
0
8K
10K
12K
16K
Cell density
FFL signal
8000
7000
6000
5000
4000
3000
2000
1000
0
8K
10K
12K
16K
Cell density

Supplementary Figure 4:

Comparison of luminescence signal detection in HeLa-Nluc-edit using Promega (A) and NanoLight Technology (B) kits. A) Nano-Glo ONE-Glo were used to measure signal generated by Nluc and FFL respectively in separated wells, whereas Dual-Glo-Nano detected FFL and subsequently Nluc signal in the same well. B) Nluc luminescence signal was detected using Nluc Assay reagents, followed by measuring of FFL (FFL Assay reagents) activity in the same well. Signals were compared from wells with cells growing at different density. Error bars represent the standard deviation values from 6 replicates.

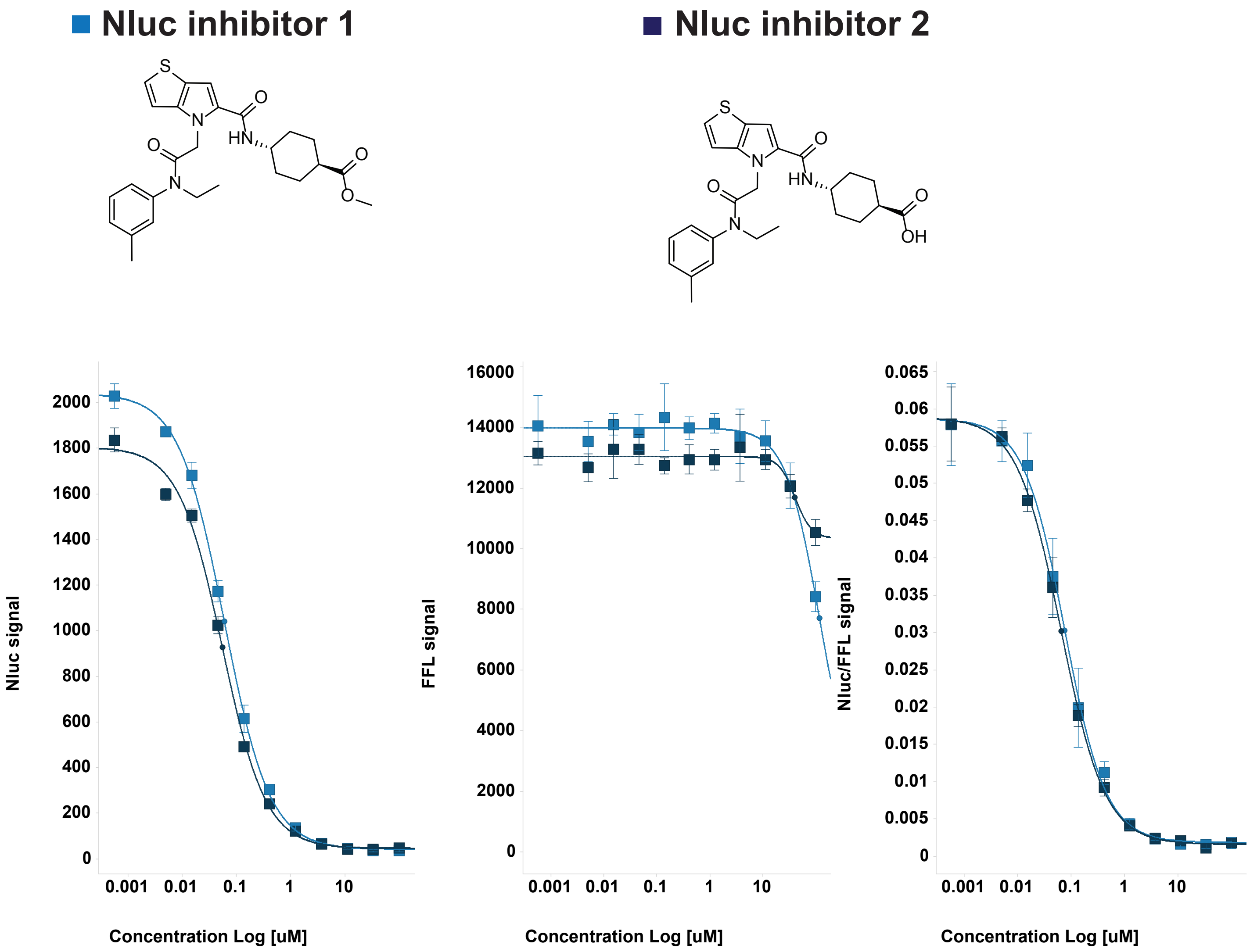
Nluc inhibitor 1
Nluc inhibitor 2
Nluc signal
FFL signal
Nluc/FFL signal
Concentration Log [uM]
0.001
0.01
0.1
1
10

Supplementary Figure 5:
Concentration response curves and structures of Nluc inhibitors. HeLa-Nluc-edit cells were treated with the inhibitors for 24h and luminescence signal was detected to calculate Nluc/FFL signal ratio. Error bars represent the standard deviation from three replicates.

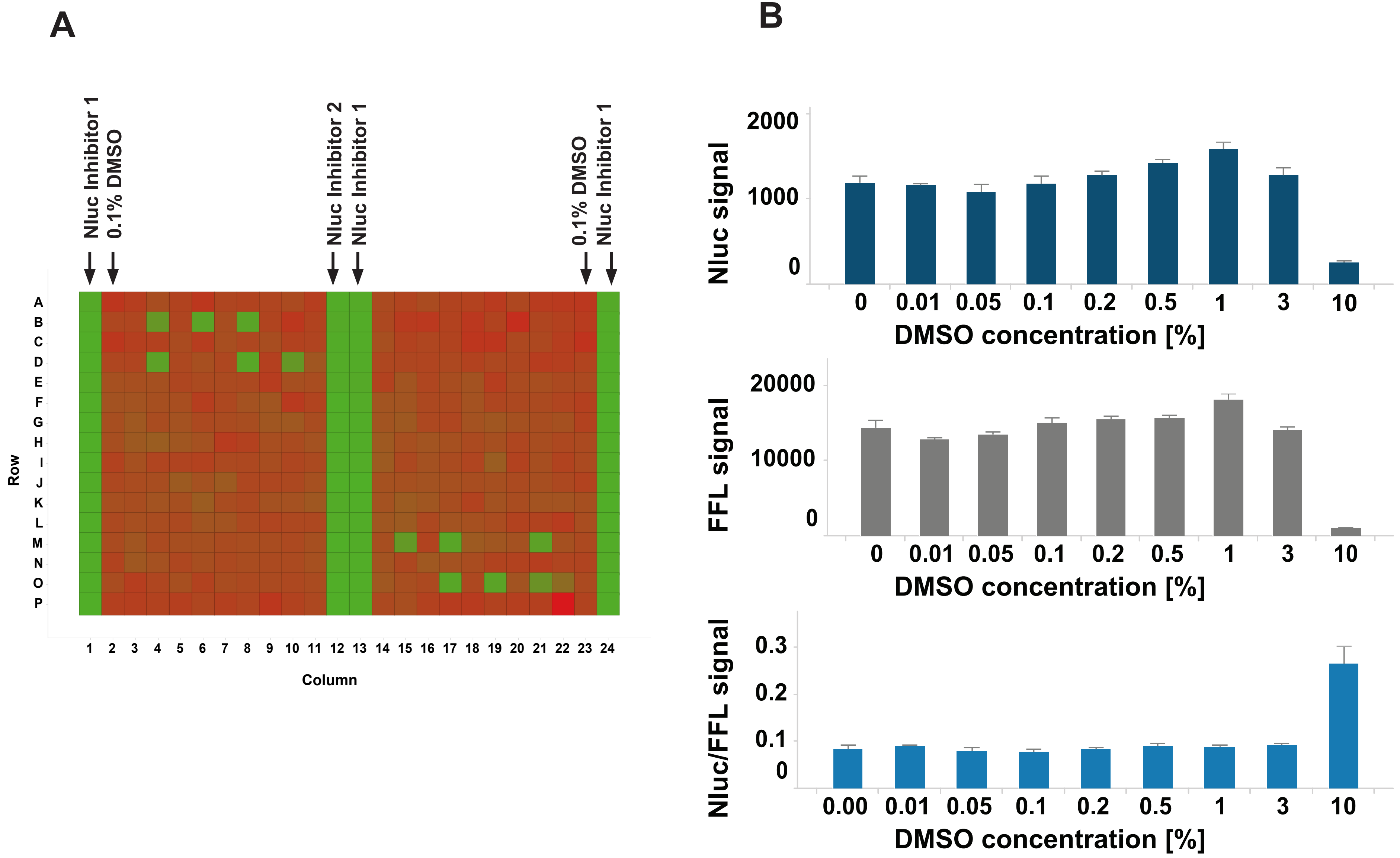

A
Nluc Inhibitor 1
0.1% DMSO
Nluc Inhibitor 2
Nluc Inhibitor 1
0.1% DMSO
Nluc Inhibitor 1
A B C D E F G H I J K L M N O P
Row
1 2 3 4 5 6 7 8 9 10 11 12 13 14 15 16 17 18 19 20 21 22 23 24
Column
B
Nluc signal
2000
1000
0
0 0.01 0.05 0.1 0.2 0.5 1 3 10
DMSO concentration [%]
FFL signal
20000
10000
0
0 0.01 0.05 0.1 0.2 0.5 1 3 10
DMSO concentration [%]
Nluc/FFL signal
0.3
0.2
0.1
0
0.00 0.01 0.05 0.1 0.2 0.5 1 3 10
DMSO concentration [%]

Supplementary Figure 6:

Validation of high throughput assay. A) Nluc/FFL signal distribution (lowest - green, highest - red) in plate. Cells in columns 1, 12 and 24 were treated with Nluc inhibitor 1 and in column 13 with Nluc inhibitor 2 at 10 μM. Negative control (0.1% DMO) was placed in columns 2 and 23. In addition inhibitors at 10 μM and 1 μM were added randomly to different wells on plate edges. B) Results of DMSO tolerance testing.

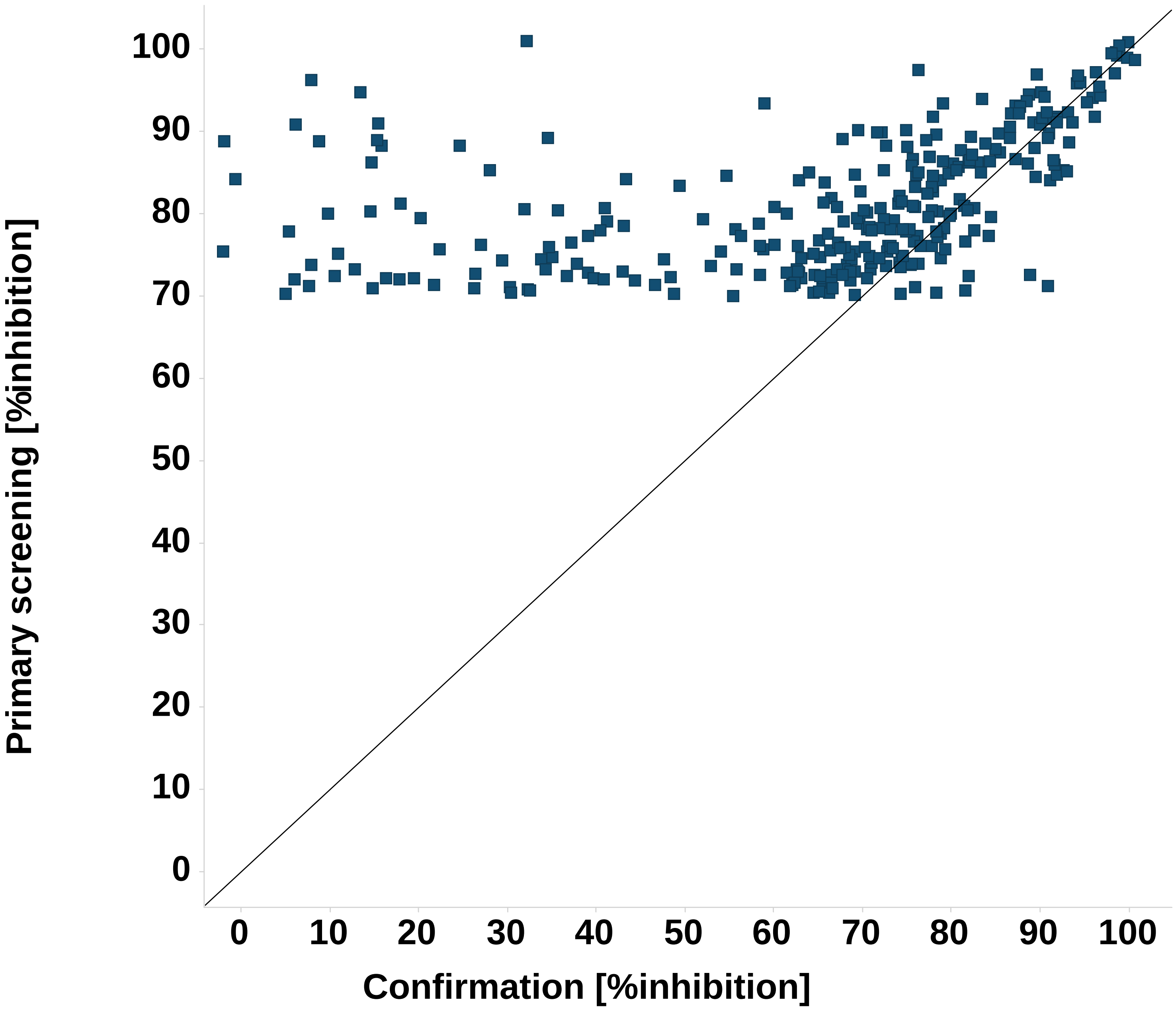
Primary screening [%inhibition]
100
90
80
70
60
50
40
30
20
10
0
0
10
20
30
40
50
60
70
80
90
100
Confirmation [%inhibition]

Supplementary Figure 7:

Comparison of confirmation (X axis) and primary screening (Y axis) results. Compounds identified as hits in primary screening (% inhibition of Nluc/FFL signal ratio ≥ 70%) were retested in independent experiment.
